# Dysfunctional mucus structure in cystic fibrosis increases vulnerability to colibactin-mediated DNA adducts in the colon mucosa

**DOI:** 10.1080/19490976.2024.2387877

**Published:** 2024-08-12

**Authors:** Amanda Mandarino Alves, Chiara Lecchi, Sharon Lopez, Alessia Stornetta, Prince P. Mathai, Peter W. Villalta, Satoshi Ishii, Emily P. Balskus, Silvia Balbo, Alexander Khoruts

**Affiliations:** a Department of Medicine, Division of Gastroenterology, Hepatology, and Nutrition, University of Minnesota, Minneapolis, MN, USA; b Department of Environmental Health Sciences, School of Public Health, University of Minnesota, Minneapolis, MN, USA; c Masonic Cancer Center, University of Minnesota, Minneapolis, MN, USA; d Department of Medicinal Chemistry, University of Minnesota, Minneapolis, MN, USA; e Department of Soil, Water, and Climate, University of Minnesota, Minneapolis, MN, USA; f BioTechnology Institute, University of Minnesota, Minneapolis, MN, USA; g Department of Chemistry and Chemical Biology, Harvard University, Cambridge, MA, USA; h Howard Hughes Medical Institute, Harvard University, Cambridge, MA, USA; i Center for Immunology, University of Minnesota, Minneapolis, MN, USA

**Keywords:** cystic fibrosis, colon cancer, colibactin, pks+ E. coli, microbiota

## Abstract

Colibactin is a recently characterized pro-carcinogenic genotoxin produced by *pks+ Escherichia coli*. We hypothesized that cystic fibrosis (CF)-associated dysfunctional mucus structure increases the vulnerability of host mucosa to colibactin-induced DNA damage. In this pilot study, we tested healthy-appearing mucosal biopsy samples obtained during screening and surveillance colonoscopies of adult CF and non-CF patients for the presence of *pks+ E. coli*, and we investigated the possibility of detecting a novel colibactin-specific DNA adduct that has not been yet been demonstrated in humans. While CF patients had a lower incidence of *pks+ E. coli* carriage (~8% vs 29%, *p* = 0.0015), colibactin-induced DNA adduct formation was detected, but only in CF patients and only in those who were not taking CFTR modulator medications. Moreover, the only patient found to have colon cancer during this study had CF, harbored *pks+ E. coli*, and had colibactin-induced DNA adducts in the mucosal samples. Larger studies with longitudinal follow-up should be done to extend these initial results and further support the development of colibactin-derived DNA adducts to stratify patients and their risk.

## Introduction

Cystic fibrosis (CF) is caused by mutations in the cystic fibrosis transmembrane conductance regulator (*CFTR*) gene, which has critical functions in epithelial ion transport and hydration of mucus. Absent or reduced CFTR expression results in thick, viscous secretions that compromise the functions of respiratory, digestive, and reproductive organs. Increased longevity of CF patients brought about by a series of advances in medical care has led to new challenges, such as gastrointestinal cancer. CF is associated with a marked increase in colorectal cancer (CRC) risk (~5–10-fold) relative to age-matched healthy controls, and this risk increases to 30-fold following organ transplantation.^1^ Similarly, we and others have reported that CF patients are prone to earlier development and progression of colon polyps, precursors of colon cancer.^2–4^ Thus, earlier colonoscopic CRC screening and more aggressive screening/surveillance intervals are recommended for CF patients relative to the general population.^5^

Over the past decade, a specific group of colibactin-producing intestinal bacteria has generated intense interest in colon cancer research. Colibactin is a non-ribosomal peptide-polyketide hybrid genotoxin, which induces inter-strand DNA crosslinks, double-strand DNA breaks, and chromosome aberrations.^6–8^ Its biosynthetic machinery is encoded in the *pks* or *clb* genomic island, which is most commonly carried by B2 phylogroup *Escherichia* coli strains but may be present in other members of Enterobacteriaceae family.^6,9,10^ Our laboratory has recently contributed to the characterization of colibactin’s chemical structure and of the corresponding specific DNA modification resulting from the reaction of the genotoxin with the *N3* position of adenine in DNA. The presence of this DNA modification (known as a DNA adduct) was confirmed in colon tissue of germ-free wild-type C57BL/6J mice inoculated with *pks+ E. coli*.^7^ Colibactin-producing *E. coli* were shown to promote colon tumorigenesis in a mouse model,^11^ and colibactin-specific mutational signatures identified in human organoids match those seen in 5–10% of the human CRCs and were found in patients genetically predisposed to colon cancer.^12–14^ Factors that weaken the mucus gut barrier increase the access of *pks+ E. coli* to the colon epithelium and contribute to epithelial injury and mucosal inflammation.^15^ Therefore, we hypothesized that defective intestinal mucus structure caused by CFTR deficiency may result in greater vulnerability to the mutagenic effects of colibactin produced by *pks+ E. coli* and may offer the opportunity to identify the colibactin-derived DNA adduct, which has never been detected in human samples before. Such an adduct would represent a chemical modification of DNA that may be a potential precursor to the colibactin-related mutations found in the signatures identified in colon cancer.

In the present work, we evaluated biopsies of endoscopically normal-appearing colon mucosa obtained during routine screening and surveillance colonoscopies of CF and non-CF patients for the presence of *pks+ E. coli*. In addition, the colon mucosa biopsies were evaluated for the presence of colibactin-derived DNA adducts previously identified in animal models using our high-resolution liquid chromatography-mass spectrometry (HRMS) method described elsewhere.^7^

## Results

### The colonoscopy findings and the carriage of pks+ E. coli in CF and non-CF patients

**Figure 1.**
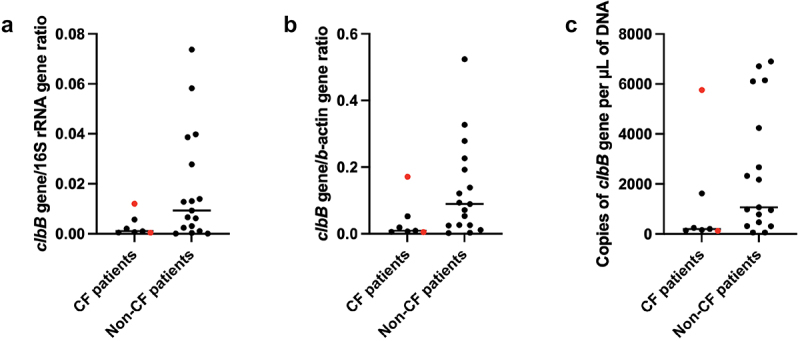
The abundance of *pks+ E. coli* in positive mucosal biopsy samples from CF and non-cf control patients was estimated by (a) ratio of *clbB* to 16S rRNA gene copies, (b) ratio of clbB to b-actin gene copies, (c) the copy number of *clbB* gene copies per µl of extracted DNA. The red squares identify samples positive for DNA adducts. The differences between CF and non-cf samples did not reach statistical significance.

Mucosal biopsies from 85 CF patients and 59 non-CF patients were tested by qPCR for the *clbB* gene to identify the presence of *pks+* bacteria (most, but not all, *pks+* bacteria are strains of *E. coli*^*10*^). The basic demographics and clinical characteristics of the two patient populations are shown in Tables 1 and 2. The CF patient cohort was somewhat younger and had greater prevalence of advanced adenomas (Table 3). Interestingly, serrated adenomas were virtually non-existent in CF patients, whereas their prevalence among adenomas was approximately 17% in non-CF patients (Table 3). *Pks+ E. coli* was present less often in CF patients relative to non-CF patients (7/85 (8.2%) versus 17/59 (28.8%); *p* = 0.0015, Fisher’s exact test). The relative burden of *pks+ E. coli* in positive samples, as measured by qPCR, was not statistically different between CF than non-CF patients (Figure 1). Detection of *pks+ E. coli* was concordant between right and left colon biopsy sites (data not shown).

**Table 1.** Demographics and disease characteristics in CF patients.

	*pks+* (*n* = 7)	*pks-* (*n* = 78)
Age, years (mean ± SD)	44.1 ± 5.2	45.6 ± 9.2
Male sex	6 (85.7%)	46 (59%)
∆508/∆508 genotype	6 (85.7%)	41 (52.6%)
Organ transplant	2 (28.6%)	23 (29.5%)
CFTR modulator (Trikafta)^a^	4 (57.1%)	44 (56.4%)
CFTR modulator (Symdeko)^b^	0	7 (9%)
Cystic fibrosis- related diabetes (CFRD)	5 (71.4%)	53 (67.9%)
Pancreatic insufficiency	7 (100%)	70 (89.7%)
History of colon cancer	1 (14.3%)	4 (5.1%)
History of colon adenomas	4 (57.1%)	39 (50%)

Note: ^a^Trikafta = elexacaftor/tezacaftor/ivacaftor.

^b^Symdeko = tezacaftor/ivacaftor.

**Table 2.** Demographics and colonoscopy indications in non-CF patients.

	*pks+* (*n* = 17)	*pks-* (*n* = 42)
Age, years (mean ± SD)	56.5 ± 7.0	53.0 ± 9.8
Sex – male	6 (35.3%)	23 (54.8%)
History of colon cancer	0	1 (2.4%)
History of colon adenomas	11 (64.7%)	21 (50%)
*Indication for colonoscopy*		
Screening	11 (64.7%)	29 (69%)
Surveillance	6 (35.3%)	11 (26.2%)
Diagnostic	0	2 (4.8%)

**Table 3.** Overview of polyp characteristics and location.

	CF pks+	CF pks-	Non-CF pks+	Non-CF pks-
Total polyps (n ± SD)^a^	1.7 ± 1.6	3.6 ± 7.4	2.9 ± 5.1	1.6 ± 2.8
Advanced polyps^a^	33.3%	22.6%	8.2%	13.4%
Serrated adenomas	8.3%	0.7%	16.3%	17.9%
*Location*^b^				
Right colon	66.7%	75.3%	61.2%	65.7%
Left colon	33.3%	19.7%	14.3%	25.4%
Indeterminate	0%	5.0%	24.5%	8.9%

Note: ^a^The number of total and advanced adenomatous polyps represents the cumulative numbers in the CF and non-CF patients in their current and prior colonoscopies.

^b^Right colon and left colon are defined as proximal and distal to the splenic flexure, respectively. Indeterminate designation was assigned when polyp location was not provided in the colonoscopy report.

Interestingly, one of the *pks+* CF patients, a 42-year-old female lung transplant recipient, was found to have colon cancer. The patient then underwent a hemicolectomy. Mucosal biopsies done during two follow-up colonoscopies since, one for colon cancer surveillance and the other for diarrhea symptoms, no longer contained *pks+ E. coli*.

### Detection of colibactin-DNA adducts in colon mucosal biopsies

In this study, DNA samples were isolated from a total of 40 tissue samples collected from CF patients and 21 samples collected from non-CF patients. This subset of samples included all the ones that were positive for *pks+ E. coli* infection. The DNA isolation process resulted in an average yield of 30 ± 22 µg. Calf thymus DNA, either spiked with the colibactin DNA adducts synthetic standards or not, was used as positive and negative controls, respectively. After undergoing neutral thermal hydrolysis, DNA was analyzed with our previously developed and reported LC-HRMS/MS method, using nanoflow chromatographic separation with nanospray ionization to achieve the highest sensitivity. The colibactin-derived DNA adducts were monitored by targeting the *m/z* 540.1772 [M+H]^+^ of the parent ion and the MS^2^ fragmentation and detection of the characteristic fragmentation ions *m/z* 229.0972 [M+H]^+^, *m/z* 387.1110 [M+H]^+^, and *m/z* 344.1057 [M+H]^+^.

**Figure 2.**
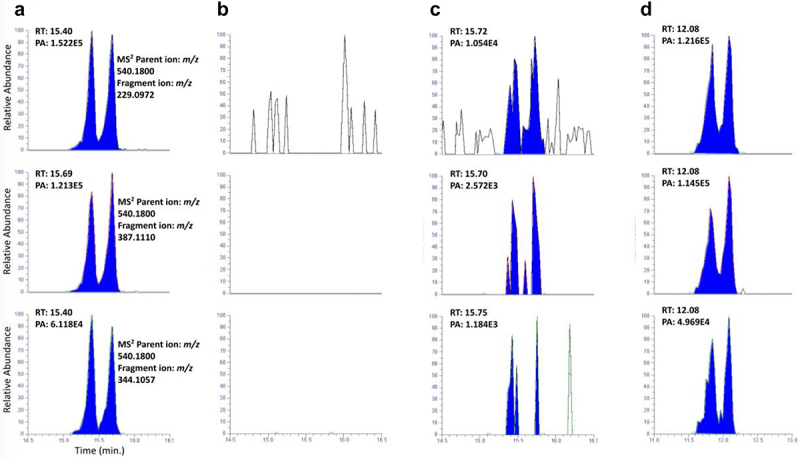
MS^2^ extracted ion chromatograms of *m/z* 540.1765 [M+H]^+^ and the fragmentation ions *m/z* 229.0972 [M+H]^+^, *m/z* 387.1110 [M+H]^+^, and *m/z* 344.1057 [M+H]^+^ obtained from the analysis of the colibactin-DNA adducts synthetic standard (panel a), a pks- non-cystic fibrosis patient (panel b), and the two pks+ cystic fibrosis subjects (panel c and d).

The colibactin-derived DNA adducts were detected in two individuals. Both were CF patients, positive for *pks+ E. coli*, recipients of lung transplants, and were not taking CFTR modulator medications. One of these patients, who was mentioned above, harbored *pks+ E. coli* at the time of her diagnosis with colon cancer and showed the highest level of the *clbB* gene among CF *pks+* patients (Figure 1). The other patient had adenomatous colon polyps and levels of the *clbB* gene that were close to the average measured in the CF *pks+* patients (Figure 1). The colibactin-derived DNA adducts were not detected in any of the other tissue samples analyzed from both the non-CF and CF participants. Additionally, the sample from the follow-up visit of the positive female cancer patient no longer contained measurable amounts of the adducts or *pks+ E. coli*, as reported above. The chromatograms obtained from the LC-MS targeted analysis of the colibactin-DNA adducts are presented in Figure 2. The presence of the analyte in the samples was confirmed by comparing the retention times and relative abundances of the observed chromatographic peaks in the fragment ion extracted chromatograms at ±5 ppm mass tolerance with those of a synthetic standard, as previously reported.

## Discussion

Ours is the first report of the detection of colibactin-derived DNA adducts in human samples, and the first to report their presence in non-neoplastic colon mucosal biopsies from human patients. The direct role in tumorigenesis played by *pks+ E. coli* was originally suggested by its overrepresentation among the mucosa-adherent bacteria in CRC tissue.^11^ In recent years, colibactin was shown to induce inter-strand cross-links via two electrophilic cyclopropane warheads that react with adenine-rich motifs in the host DNA.^6–8^ Exposure of human intestinal organoids to *pks+ E. coli* induces a colibactin-associated mutational signature, and the same mutational signature can be detected in a subset of human colon cancers.^12,14^ Furthermore, human organoids recovered from short-term infection with *pks+ E. coli* demonstrate Wnt-independence,^16^ which is characteristic of most CRC cells due to loss of Wnt-mediated regulation of the adenomatous polyposis coli (*APC*) pathways. Notably, the *APC* gene is preferentially targeted by colibactin mutagenesis in CRC tissues.^13^

It is tempting to speculate that changes in the gut microbiome, including the increasing prevalence of *pks+ E. coli*,^17^ contribute to the rising incidence of colon cancer among the younger patient populations.^18^ However, the mere presence of *pks+ E. coli* is not sufficient to drive adenoma and colon cancer formation. Interestingly, our findings support this observation, showing detectable levels of the colibactin-derived DNA adducts only in samples from 2 CF patients with specific characteristics and not in all the samples containing *pks+ E. coli*. This result suggests the absence of a simple correlation between the abundance of *pks+ E. coli* and detectable levels of the colibactin-derived DNA adducts, and therefore a potential for these adducts to be used to detect susceptibility to specific cancer risk. Carcinogenic potential of colibactin requires penetration of the mucosal barrier and direct contact with the host epithelial cells. A defective mucosal barrier is characteristic of inflammatory bowel disease, which is a known risk factor for CRC, and neoplastic tissue itself. In the current study, we hypothesized that CF patients are more vulnerable to colibactin exposure due to their dehydrated and dysfunctional mucus structure. It is notable that both CF patients with the adducts were recipients of lung transplants and consequently were not taking CFTR modulator medications typically used to improve lung function. The potential benefits of CFTR modulator therapies on the extra-pulmonary sites compromised by CF remain unexplored at this time.

A potentially important aspect of carcinogenesis in the colon is polymicrobial biofilm formation. Invasive polymicrobial bacterial aggregate structures have been associated with the majority of right-sided CRCs and tumor-free mucosa distant from their tumors.^19^ Such biofilms have been shown to have high abundances of *pks+ E. coli* and enterotoxigenic *Bacteroides fragilis*. ^20^ Degradation of protective mucus by enterotoxigenic *B. fragilis* can enhance contact of *E. coli* with the host epithelial cells and increase delivery of colibactin. Biofilm formation contributes to CF-associated infections in the lungs,^21,22^ but thus far little attention has been given to biofilms in the intestine of CF patients. Interestingly, the presence of *pks+ E. coli* in the biofilms in colon biopsies from patients with ulcerative colitis was associated with the development of dysplasia, an early lesion in the progression of carcinogenesis.^23^

The rate of carriage of pks+ *E. coli* in our non-CF patient cohort was comparable to that seen in other studies of Western patients.^24^ However, the presence of *pks+ E. coli* was lower among the CF patients. It is possible that higher burden of antibiotics experienced by CF patient may be contributing to this finding. The burden of *pks+ E. coli* is higher among patient populations characterized by increased relative abundances of Proteobacteria in their gut microbiome, e.g., patients with inflammatory bowel diseases and patients with recurrent *Clostridioides difficile* infections.^24,25^ Previously, others and we did not find an increased relative abundance of Proteobacteria in the gut microbiome of CF patients.^26,27^ Our results also suggest that the presence of *pks+ E. coli* can be transient. One explanation for this may be greater exposure to antibiotics that target Proteobacteria. The dynamics of intestinal colonization by *pks+ E. coli* needs to be studied in future investigations.

Our data, while still anecdotal, suggests a need for further investigations of the potential effects of the CFTR modulator medications in the gastrointestinal tract, and their potentially chemoprotective role against colon cancer. Specifically, a clear understanding is necessary of the impacts that the levels of exposure, duration, and conditions for an infection with *pks+ E. coli* have on the corresponding colibactin-derived DNA adduct levels. Furthermore, the investigation of the persistence of the adduct in DNA, its susceptibility to the action of DNA repair mechanisms, and its mutagenic potential contributing to the later development into the identified colibactin mutational signatures, require additional attention. Our findings need to be further confirmed by extending these analyses to a larger number of samples. Additionally, an improvement in our DNA adduct detection methodology is needed to make more quantifiable measurements and more carefully determine our method’s sensitivity and limit of detection. Thus, the two samples positive for the colibactin-derived DNA adducts had amounts of DNA that were greater than 40 μg, while most of the negative samples had lesser amounts. Further studies will focus on verifying the minimum amount of DNA necessary to detect these DNA modifications. Finally, we focused our attention on one microbiota-derived pro-carcinogenic genotoxin, colibactin, and its highly specific DNA adduct, due to the fact that to our knowledge, no other biosynthetic pathways that create a similar electrophile have been identified in bacteria so far. The *pks/clbB* genes are found in other gut bacteria beyond *E. coli*, but the biosynthetic genes are very distinctive, and there is no other NRPS-PKS pathway in any gut bacteria that looks similar to the one producing colibactin. However, it is possible that other microbiota-derived toxins capable of generating pro-carcinogenic DNA adduct formation are yet to be discovered and characterized, although the therapeutic focus on improving the gut barrier function would be applicable to all.

## Patients, materials/methods

### Patient samples

Mucosal biopsies were obtained in patients undergoing routine screening and surveillance colonoscopies at the University of Minnesota. The majority of CF patients receiving their medical care at the Minnesota Cystic Fibrosis Center participate in the dedicated, systematic colonoscopic CRC screening program, as described previously.^2^ The mucosa targeted by biopsies was endoscopically normal and did not include any polyp tissue. Two pinch biopsies from the ascending colon were placed into Eppendorf tubes without any liquid medium, immediately frozen on dry ice, and transferred to −80°C or −20°C freezers for analysis. Additional two pinch biopsies were obtained from the ascending and descending colon and placed into separate test tubes containing 95% ethanol for microbiome studies. All biopsies were obtained using the Radial Jaw 4 Jumbo w/Needle 240 (length) forceps for a 3.2 mm working channel (Boston Scientific, Marlborough, MA; Catalog # M00513371). The protocol was approved by the University of Minnesota Institutional Review Board.

### Materials and chemicals

Reagents for DNA isolation were purchased from QIAGEN Sciences (Germantown, MD). Water (LC-MS grade), acetonitrile (ACN, LC-MS grade), 2-propanol (IPA, LC-MS grade), and formic acid (FA, 98% v/v) were purchased from Fisher Scientific (Hanover Park, IL, USA). Chloroform was obtained from Sigma-Aldrich (St. Louis, MO, USA). Calf thymus DNA (CT-DNA, 5 mg), Tris base, single-filtration membrane filters (Microcone,10 kDa cutoff, 0.5 mL) were purchased from Millipore Sigma (St. Louis, MO, USA). Cell lysis solution, protein precipitation solution, RNase A, and proteinase K were obtained from Qiagen (Hilden, Germany). Polypropylene vials (0.3 mL) were purchased from Thermo Scientific (Hanover Park, IL, USA). Synthetic standard of the colibactin-derived DNA adduct (C_23_H_25_N_9_O_5_S, *m/z* 540.1772 [M+H]^+^) was obtained as previously described.^7^

### Detection of the pks island in mucosal biopsies

The presence of the *pks* island was detected by qPCR targeting the *clbB* gene of *E. coli*. The primer set included 5’-GCGCATCCTCAAGAGTAAATA-3’ as the forward primer and 5’-GCGCTCTATGCTCATCAACC-3’ as the reverse.^28^ qPCR was done using the StepOnePlus Real-Time PCR System (Applied Biosystems, Foster City, CA, USA). The reaction mixture included a total of 20 µL and consisted of 1× iTaq Universal SYBR Green Supermix (Bio-Rad, Hercules, CA, USA), 0.6 uL each of 10 µmol/L forward and reverse primers, and 5 µL of the DNA diluted 1:10. Cycling conditions are as follows: 95°C for 10 min, 95°C for 15 sec, and 60°C for 60 sec. Analysis of the melt curve was done after each PCR to determine specificity. Pks data was normalized with β-actin (forward: 5’-TCCGCAAAGACCTGTACGC-3’, reverse: 5’-CATTGTAGCACGTGTGTAGCC-3’)^1^ and 16S rRNA (forward 515: 5’-GTGCCAGCMGCCGCGGTAA-3’, reverse 806: 5’-GGACTACHVGGGTWTCTAAT-3’) genes.^29^ β-actin and 16S qPCR were done using a QuantStudio 5 (Applied Biosystems, Foster City, CA, USA). 400 nM of primers were used for both β-actin and 16S, with cycling conditions of 95°C for 10 min, 95°C for 15 sec, and 58°C for 30 sec for β-actin, and 95°C for 10 min, 95°C for 15 sec, 50°C for 30 sec, and 72°C for 30 sec for 16S.

gBlock standards were included in each run with a range from 10^8^ to 10^1^ gene copies per µL (Integrated DNA Technologies, Coralville, IA, USA), as were no-template controls. The threshold cycle (Ct) value and the standard curves were determined by the StepOnePlus software v2.3. Amplification efficiencies ranged from 92.4% to 98.0% for *clbB*, 90.6% to 91.2% for β-actin, and 95.5% to 97.8% for 16S rRNA genes. The gene copies were calculated from the Ct values using the standard curves of the assay, and values below the lowest concentration on the standard curve were considered negative.

### DNA isolation and purification for DNA adducts analysis

The amount of tissue obtained with the biopsies (~5–50 mg) provided sufficient material for DNA adducts analysis (~30 µg). DNA was isolated following a tissue DNA purification protocol (Gentra Puregene Tissue Kit Qiagen, Hilden, Germany). Briefly, the frozen tissues were weighed, minced, and homogenized after adding 300 μL of Cell Lysis solution into a 2 mL micro-centrifuge tube (DNA LoBind). Then, the samples were incubated with 2 μL of Protein Proteinase K overnight with gentle shaking at room temperature. RNAse A Solution (2 μL) was added the next day to the cell lysates and incubated at room temperature for 2 hours in the shaker. Protein Precipitation Solution (100 μL) was added and mixed by vortexing vigorously, and then centrifuged at 16,000 × g for 3 min at 4°C. The supernatants were transferred into 2 mL clean micro-centrifuge tube containing 400 μL of ice-cold isopropanol, and after inverting several times, the DNA became visible as white strands. Then, the samples were centrifuged for 1 minute at 16,000 × g at 4°C and the supernatants were carefully discarded. The DNA was then dissolved in 300 μL of 10 mM Tris-HCl buffer containing 1 mM EDTA (pH 7.0) and further purified with 300 μL of chloroform/isoamyl alcohol (24:1). After centrifuging at 16,000 × g for 5 min at 4°C, the upper aqueous layers were carefully collected and transferred into clean 2 mL micro-centrifuge tubes containing 30 μL of 5 M NaCl solution. Then, the DNA was precipitated by adding 300 μL of ice-cold isopropanol. The DNA pellets were washed with ice-cold 70% isopropanol and 100% isopropanol sequentially, centrifuging the samples at 16,000 × g for 3 min at 4°C and discarding the supernatant each time. Finally, the DNA pellets were allowed to air dry for up to 15 min and stored at −20°C for future use.

### DNA hydrolysis and sample preparation for LC-MS

The DNA pellets were dissolved in 100 μL of LC-MS water, and the amount of DNA was estimated by measuring the concentration by UV/Vis spectrometry using the absorbance optical mode and monitoring the 260 and 280 nm wavelengths. The samples were transferred into 300 μL polypropylene vials and heated at 80°C for 1 h for neutral thermal hydrolysis. The hydrolyzates were then filtered with pre-washed Microcon centrifugal filters (10 K MW cutoff) at 12,000 × g for 45 min at room temperature. Finally, the filtrates were transferred in new 300 μL polypropylene vials and brought to complete dryness in the SpeedVac. The samples were stored at −20°C prior to mass spectrometric analysis.

### Chromatography for LC-MS

Mass spectrometric data was acquired with the following conditions. Each dried sample was reconstituted in 10 μL of H_2_O and 5 μL of sample, corresponding to 5–50 μg of DNA, was injected onto an UltiMate 3000 RSLCnano UPLC (Thermo Scientific, Waltham, MA) system equipped with a 5 μL injection loop. Separation was performed with a capillary column (75 μm ID, 20 cm length, 10 μm orifice) created by hand packing a commercially available fused-silica emitter (New Objective, Woburn, MA) with 5 μm Luna C18 bonded separation media (Phenomenex, Torrance, CA). The flow rate was 1000 nL/min for 5.5 min, then decreased to 300 nL/min with a 15 min linear gradient from 2 to 50% CH_3_CN in 0.05% formic acid aqueous solution, an increase to 98% CH_3_CN in 1 min, with a 2 min hold and a 3 min re-equilibration at 1000 nL/min at 2% CH_3_CN. The injection valve was switched at 6 min to remove the sample loop from the flow path during the gradient.

### Mass spectrometry

Mass spectrometric data was acquired using a Lumos Orbitrap Tribrid mass spectrometer (Thermo Scientific, Waltham, MA). Positive mode electrospray ionization was used under nanospray conditions (300 nL/min) with a Thermo Scientific Nanoflex ion source, spray voltage of 2.2 kV, capillary temperature of 300°C, and an S-Lens RF level setting of 40%. Targeted MS^2^ detection was acquired with fragmentation of 540.2 m/z, a quadrupole isolation window of 1.5 m/z, and an HCD fragmentation energy of 25%. Additional parameters were a resolution setting of 120,000, an AGC setting of 1 × 10^6^, and a maximum injection time of 1000 ms.

## Statistical analysis

Statistical analysis was done using GraphPad Prism 9.5.1 software. Fisher’s exact test was used to determine if there are nonrandom associations between categorical variables. An independent samples t-test was employed to compare the means between two distinct groups. The Mann-Whitney U test was utilized to compare distributions of independent groups when the assumptions for parametric tests were not met.

## Data Availability

The raw data containing clinical information and *pks* qPCR results was deposited with Harvard Dataverse, V1 (https://doi.org/10.7910/DVN/BIT4CP).
